# The American Pharmaceutical Association

**Published:** 1882-10

**Authors:** 


### The American Pharmaceutical Association.

The thirteenth annual meeting of this Association was held at Niagara Falls, Sept. 12 to 15, and in point of attendance and general interest it exceeded any of its predecessors. The presence of the fair sex was particularly noticeable, most of the members being accompanied by their wives, sisters, cousins or aunts, and as all were decorated with a badge on which a gold mortar was conspicuous, the mortar-lity at Niagara Falls during their session was striking.

The initial session of the organization was held in the afternoon and was occupied by organization and the reading of the President's address. The mornings of the two following days were devoted to business and the afternoons and evenings to sight seeing and entertainments. Several good papers were presented, noticeably those of Prof. Bedford, of New York, and Wm. Saunders, of London, Ont., but the dearth of papers of any real pharmaceutical scientific interest was noticeable. The exhibition of pharmaceutical products was exceptionally good, as will be seen from the following interesting communication from our correspondent:

It would sometimes seem as if in the earnest desire to pursue investigations in pathology and the natural history of disease, so fascinating to the physician with scientific taste, that we are in danger of losing sight of the importance of drugs and their effect on the animal economy in disease.

For some time past therapeutic nihilism has been fashionable. To this many causes have perhaps contributed, but for our present purpose it is only necessary to mention one, and that is the past unreliability of drugs.

It is becoming apparent that this want of faith in the efficacy of drugs is passing away, and indeed he who would accomplish much by their aid must earnestly believe in them himself.

Patients are quick to detect, even in the manner of the prescriber, his lack of absolute confidence in the medicine used. The pharmaceutist must after all be our ultimate reliance, and it would be well perhaps if we stopped more frequently to inquire what is being done by them, to provide us with reliable, convenient and more palatable medicines.

These thoughts are suggested by a recent visit to the handsome display of "The American Pharmaceutical Association," at Niagara Falls.

Possibly a brief description of some of the exhibits may not be uninteresting to your readers. In the centre of the room was the artistic display of chemicals from the well-known house of Powers & Weightman. The reputation of this house is well established, and they always make a beautiful display. Pills on the right of you; pills on the left of you; pills in front of you; pills, white, red, yellow, brown, black; pills, gelatine coated, sugar coated; pills, compressed; pills! until one wonders if we are not intended to be *fed* on pills; and we echo the remark at our elbow: "At last pills look good enough to eat!" We have no disposition to discuss the relative merits of different coatings, or of any coating, or whether pills should be manufactured in this wholesale way. The fact that they are manufactured in immense quantities and dispensed by physicians seems to indicate a demand that cannot be suppressed. Our patients will not swallow bulky or unpalatable pills, and the important point, after all, is that they are as represented.

In the absence of a Government inspector instances of fraud should be ruthlessly exposed and published far and wide.

In the exhibit of W. H. Schieffelin & Co. we noticed a number of original packages of opium, cajeput oil, etc., and some Chinese drugs with unpronounceable names, evidencing the enterprise of this widely-known firm. Some fine samples of *genuine* castile soap, an old-fashioned and useful article—quite difficult to find pure now—also samples of oleates, etc.

John Wyeth & Bro., of Philadelphia, were represented with a full assortment of their compressed pills, and, amongst the novelties, Quinquinia, "a natural combination of the alkaloids of cinchona bark," claiming to be cinchona bark, minus its woody fibre and resins, and not so apt to induce cerebral excitement, grain for grain as efficient as quinine, highly recommended and deserving of a trial.

Among the newer preparations the "Dialysates" of McIntyre & Emburg, of New York, deserve mention, claiming uniformity of strength (one grain of the pure drug to two grains of the dialysate) and freedom from precipitation and cloudiness when mixed with water, syrup, etc.

Seabury & Johnson made a fine display of their excellent plasters, now well known and justly valued for their convenience. They also show Lister's gauze, made with carbolic acid, eucalyptus, and iodoform. This energetic and enterprising firm make a full line of materials for Lister's dressing of excellent quality. Among their convenient novelties is isinglass plaster, on Canton flannel.

Thorp & Lloyd Brothers displayed, apparently, podophylin enough to purge the whole population. They manufacture, largely, different resins and alkaloids. Amongst others we noticed a fine sample of white alkaloid of hydrastis.

T. W. Heinemann & Co., of Chicago, made a fine exhibit of their specialty in water-proof plaster, preserving the pliability of the fabric wonderfully; for those esthetically inclined they make a water-proof court plaster on satin! Their mustard on muslin seems well adapted for the ready and convenient rubefacient action.

In pharmaceutical apparatus, the Porpapine Pill Coating Machine of Charles C. Wells, of Saratoga, is a novelty, we should think admirably adapted for the purpose of enabling the dispensing druggist to rapidly and easily coat pills. It is certainly ingenious, and deserving investigation by druggists. Those who have tried the manufacture of butter of cocao suppositories will welcome the suppository machine of A. M. Knowlson, by which rectal and urethral suppositories can be quickly made, the chief objection to it, however, being the high price.

Space will not admit of mention of the details of all the exhibits, but the whole display was highly creditable, and shows that the pharmacists are alive and awake, and anxious to meet every want of the medical profession.

One of the meetings of the association was made especially interesting by the visit of Prof. Carpenter, the distinguished physiologist of London, who gave an interesting account of the microscope and its development.

The officers for the ensuing year are as follows: President, Charles A. Heintsch, Lancaster, Pa.; First Vice-President, John Ingalls, Macon, Ga.; Second Vice-President, Louis Dohme, Baltimore; Third Vice-President, William B. Blanding, Providence; Treasurer, Charles A. Luffts, Dover, N. H.; Permanent Secretary, John M. Maisch, Philadelphia; Reporter on Progress of Pharmacy, C. Lewis Diehl, Louisville.

